# A Conserved C-Terminal Domain of the *Aspergillus fumigatus* Developmental Regulator MedA Is Required for Nuclear Localization, Adhesion and Virulence

**DOI:** 10.1371/journal.pone.0049959

**Published:** 2012-11-21

**Authors:** Qusai Al Abdallah, Se-In Choe, Paolo Campoli, Stefanie Baptista, Fabrice N. Gravelat, Mark J. Lee, Donald C. Sheppard

**Affiliations:** 1 Department of Microbiology and Immunology, McGill University, Montreal, Quebec, Canada; 2 Department of Medicine, McGill University, Montreal, Quebec, Canada; University of Wisconsin - Madison, United States of America

## Abstract

MedA is a developmental regulator that is conserved in the genome of most filamentous fungi. In the pathogenic fungus *Aspergillus fumigatus* MedA regulates conidiogenesis, adherence to host cells, and pathogenicity. The mechanism by which MedA governs these phenotypes remains unknown. Although the nuclear import of MedA orthologues has been reported in other fungi, no nuclear localization signal, DNA-binding domain or other conserved motifs have been identified within MedA. In this work, we performed a deletion analysis of MedA and identified a novel domain within the C-terminal region of the protein, designated MedA^346–557^, that is necessary and sufficient for nuclear localization of MedA. We further demonstrate that MedA nuclear localization is required for the function of MedA. Surprisingly, expression of the minimal nuclear localization fragment MedA^346–557^ alone was sufficient to restore conidogenesis, biofilm formation and virulence to the *medA* mutant strain. Collectively these results suggest that MedA functions in the regulation of transcription, and that the MedA^346–557^ domain is both necessary and sufficient to mediate MedA function.

## Introduction

*Aspergillus fumigatus* is a saprophytic fungus that plays an important ecological role in recycling organic material [1], [2]. It is also the primary causative agent of invasive aspergillosis, a fungal infection of immunocompromised patients with both high mortality and morbidity [3], [4]. *A. fumigatus* depends largely upon its asexual life cycle to generate spores, termed conidia, for propagation and dissemination [5], [6]. Like other *Aspergillus* species, the asexual life cycle of *A. fumigatus* has distinct developmental stages [7], [8]. Initially, airborne conidia land upon a suitable substrate and germinate to form tubular hyphae which grow and branch to form a network of mycelium. The mycelia expand indefinitely forming a radially symmetric colony. Once the hyphae mature, multicellular conidiation structures are formed which can produce large numbers of conidia to allow the cycle to continue [9], [10].

**Table 1:** Oligonucleotides used in this study.

Name	Sequence (5' → 3')
MedA-F	ATG GAT ATC ATG TCG ACT TTT CAG AAA CCC CCG
MedA-R	ATA ATA CGC GGC CGC ACA CCC GTG GGA AGG
AlcAp-For	AGA AGA GAG CTC TGT ACC GGT TGA AAA GCT GAT TGT G
AlcAp-Rev	ATA ATA GCG GCC GCG ATA TCC ATT TTG AGG CGA GGT G
**Truncation of MedA:**	
MedA736-F	ATT ATG GAT ATC GCT GCA GGA CAG CAG GGA TAT GA
MedA892-F	ATT ATG GAT ATC CAC TCG TCC ATT CGA GCA CCT TCT CCT
MedA1036-F	ATT ATG GAT ATC AAT CCG ACG CTT ATT CGG ACG TCG ACC
MedA1045-F	CTC AAA ATG GAT ATC CTT ATT CGG ACG TCG ACC TT
MedA1647-R	CTC ACC ATC GCG GCC GCG GAA CTG ATT GGC GTT
MedA-1671-R	ATA ATA ATA GCG GCC GCC GCT GCT CCA TTG CCA GC
**Site-directed mutagenesis:**	
NLS1-F	AAG ACT CTC CCT CCG ATG AAT CGG CTG CAG GAC AGC AGG GAT ATG
NLS1-R	CAT ATC CCT GCT GTC CTG CAG CCG ATT CAT CGG AGG GAG AGT CTT
NLS2-F	CTC TAT GGC GGA GGG ATG GAC ACT GGT TCA ATT CAC TCG CAT GCA
NLS2-R	TGC ATG CGA GTG AAT TGA ACC AGT GTC CAT CCC TCC GCC ATA GAG
NLS3-F	CGT CCG TTT CAC CGT GGA GGA GGC AGA CAG TGA AGA CTT CTT CAA
NLS3-R	TTG AAG AAG TCT TCA CTG TCT GCC TCC TCC ACG GTG AAA CGG ACG
NLS4-R	ATA AGT ACT TAC GTA GGG GGC GGG AAA GCC CAT AAT A
**Phenotype Complementation:**	
PmedA-F	CCG CCG CGC ACC CTC ACA TTC GCG AAG ATC TTC AAA GTG GGA CTC GGC TT
MedA-NheI-R	TCG TAA GAG CTA GCG TAC TCC TGA TTT GGC AG
PM(1036)-F	GTC TGA GCC CTT CCT GTC TAT GTC GAT CAA TCC GAC GCT TAT TCG GAC GT
PM(1036)-R	ACG TCC GAA TAA GCG TCG GAT TGA TCG ACA TAG ACA GGA AGG GCT CAG AC
PmedA-AgeI-F	ATA TGA TGA CCG GTT CAA AGT GGG ACT CGG CTT
**RT-PCR:**	
RT-MedA-F	GGC ACT CGC AAT CAT TCA ATC CGT ATG
RT-MedA-R	GTG GAT TGT GCT TCC GTT CTG CAT G
TEF1-sense	CCA TGT GTG TCG AGT CCT TC
TEF1-antisense	GAA CGT ACA GCA ACA GTC TGG

Conidiation in *Aspergillus* species has been studied extensively in the model organism *Aspergillus nidulans* and is regulated via the key core regulatory proteins BrlA, AbaA and WetA. Another regulatory protein, MedA, was identified as a temporal modifier of the expression of these core conidiation proteins [11]–[15]. Mutations in the *A. nidulans medA* gene resulted in abnormal and reduced conidiogenesis [11], [16]. In other fungi, orthologues of MedA have been found not only to govern asexual reproduction, but also to play a role in virulence. For example, deletion of the *A. fumigatus medA* gene resulted in a strain with impaired biofilm production and a reduced capacity to adhere to pulmonary epithelial cells, endothelial cells and fibronectin *in vitro* [17]. The Δ*medA* mutant also exhibited attenuated virulence in an invertebrate and murine model of invasive aspergillosis, suggesting that the downstream targets of *A. fumigatus* MedA mediate virulence [17]. Disruption of ACR1, the *Magnaporthe grisea* orthologue of *medA*, resulted in the production of conidia that fail to cause disease [18]. Similarly, Ren1, the MedA orthologue in *Fusarium oxysporum*, is essential for microconidia and macroconidia development and for the correct differentiation of conidiophores and phialides [19]. However, unlike in *A. fumigatus* and *M. grisea*, the *REN1* deletion mutant of *F. oxysporum* was fully virulent [19].

Previous studies have hypothesized that MedA, Acr1 and Ren1 are transcription regulators that govern fungal conidiogenesis and adherence to substrates. In support of this hypothesis, transcriptome studies in *A. fumigatus* have identified the dysregualtion of over 142 genes in the *medA* deletion mutant (unpublished data). However, no DNA-binding domains or other conserved motifs have been identified within MedA or its homologues. Although two studies have reported nuclear localization of GFP-tagged *A. nidulans* MedA and *F. oxysporum* Ren1, a nuclear localization signal (NLS) has not been identified within these proteins, and the role of nuclear localization in governing asexual reproduction, adherence or virulence is unknown [19], [20].

In this work, we hypothesized that nuclear localization is required for the function of MedA. To test this hypothesis, we performed a deletion and mutational analysis of *A. fumigatus* MedA. We identified a conserved domain located within the C-terminal portion of MedA which mediates nuclear localization. Further, we demonstrate that nuclear localization of this domain is both necessary and sufficient for MedA regulation of asexual reproduction, adherence and virulence.

**Table 2:** Plasmids used in this study, combination of oligonucleotides, and the corresponding expression strains of *A. fumigatus.*

MedA designation	Plasmids	Genotype	Oligonucleotides	Strain	Reference
–	pAL4	*Amp^R^; pyr-4; alcA(p)*	–	–	[44]
–	pGFP-Phleo	*Amp^R^; Phleo^R^; gpdA(p)-egfp*	–	–	[31]
–	pMedA-GFP	*Amp^R^; Phleo^R^; gpdA(p)-medA-egfp*	MedA-F & MedA-R	–	This study
**Truncation of MedA:**					
MedA^1–683^	pAlcA-MedA-GFP	Amp^R^; *Phleo^R^; alcA(*p)-*medA-egfp*	AlcAp-For & AlcAp-Rev	WT-Mgfp	This study
MedA^245–683^	pMedA(736–2049)	*Amp^R^; Phleo^R^; alcA(p)-medA^245–683^-egfp*	MedA736-F & MedA-R	WT-M(736–2049)	This study
MedA^298–683^	pMedA(892–2049)	*Amp^R^; Phleo^R^; alcA(p)-medA^298–683^-egfp*	MedA892-F & MedA-R	WT-M(892–2049)	This study
MedA^346–683^	pMedA(1036–2049)	*Amp^R^; Phleo^R^; alcA(p)-medA^346–683^-egfp*	MedA1036-F & MedA-R	WT-M(1036–2049)	This study
MedA^349–683^	pMedA(1045–2049)	*Amp^R^; Phleo^R^; alcA(p)-medA^349–683^-egfp*	MedA1045-F & MedA-R	WT-M(1045–2049)	This study
MedA^1–557^	pMedA(1–1671)	*Amp^R^; Phleo^R^; alcA(p)-medA^1–557^-egfp*	MedA-F & MedA1671-R	WT-M(1–1671)	This study
MedA^1–549^	pMedA(1–1647)	*Amp^R^; Phleo^R^; alcA(p)-medA^1–549^-egfp*	MedA-F & MedA1647-R	WT-M(1–1647)	This study
MedA^346–557^	pMedA(1036–1671)	*Amp^R^; Phleo^R^; alcA(p)-medA^346–557^-egfp*	MedA1036-F & MedA1671-R	WT-M(1036–1671)	This study
MedA^349–549^	pMedA(1045–1647)	*Amp^R^; Phleo^R^; alcA(p)-medA^349–549^-egfp*	MedA1045-F & MedA1647-R	WT-M(1045–1647)	This study
**MedA NLS Site-directed mutagenesis:**					
MedA^ΔNLS1^	pMedA-GFP-ΔNLS1	*Amp^R^; Phleo^R^; alcA(p)-medA^ΔNLS1^-egfp*	MedA-F, MedA-R, NLS1-F & NLS1-R	WT-Mgfp-ΔNLS1	This study
MedA^ΔNLS2^	pMedA-GFP-ΔNLS2	*Amp^R^; Phleo^R^; alcA(p)-medA^ΔNLS2^-egfp*	MedA-F, MedA-R, NLS2-F & NLS2-R	WT-Mgfp-ΔNLS2	This study
MedA^ΔNLS3^	pMedA-GFP-ΔNLS3	*Amp^R^; Phleo^R^; alcA(p)-medA^ΔNLS3^-egfp*	MedA-F, MedA-R, NLS3-F & NLS3-R	WT-Mgfp-ΔNLS3	This study
MedA^ΔNLS4^	pMedA-GFP-ΔNLS4	*Amp^R^; Phleo^R^; alcA(p)-medA^ΔNLS4^-egfp*	MedA-F & NLS4-R	WT-Mgfp-ΔNLS4	This study
**Phenotype Complementation:**					
MedA	pPmedA-MedA-GFP	*Amp^R^; Phleo^R^; medA(p)-medA-egfp*	PmedA-F & MedA-NheI-R	ΔM-Mgfp	This study
MedA^ΔNLS1^	pPmedA-MedA-GFP-ΔNLS1	*Amp^R^; Phleo^R^; medA(p)-medA^ΔNLS1^-egfp*	-	ΔM-Mgfp-ΔNLS1	This study
MedA^ΔNLS2^	pPmedA-MedA-GFP-ΔNLS2	*Amp^R^; Phleo^R^; medA(p)-medA^ΔNLS2^-egfp*	-	ΔM-Mgfp-ΔNLS2	This study
MedA^ΔNLS3^	pPmedA-MedA-GFP-ΔNLS3	*Amp^R^; Phleo^R^; medA(p)-medA^ΔNLS3^-egfp*	-	ΔM-Mgfp-ΔNLS3	This study
MedA^ΔNLS4^	pPmedA-MedA-GFP-ΔNLS4	*Amp^R^; Phleo^R^; medA(p)-medA^ΔNLS4^-egfp*	-	ΔM-Mgfp-ΔNLS4	This study
MedA^346–557^	pPmedA-MedA(1036–1671)	*Amp^R^; Phleo^R^; medA(p)-medA^346–557^-egfp*	PmedA-AgeI-F, PM(1036)-R, PM(1036)-F, & MedA-1671-R	ΔM-M(346–557)	This study

## Materials and Methods

### Oligonucleotides, Fungal Strains and Media

The oligonucleotides used are fully described in Table 1. The *Aspergillus fumigatus* strain Af293 [21] was kindly provided by P. Magee (University of Minnesota, St. Paul, MN). All *A. fumigatus* strains were generated in this study by ectopic integration of plasmids. Transformation of *A. fumigatus* was achieved using protoplasting [17]. Conidia were harvested from mycelia grown for 6 days at 37°C on YPD agar (1% yeast extract, 2% peptone, 2% glucose, and 1.5% agar, pH 6.5) using PBS supplemented with 0.1% (v/v) Tween 80, pH 7.4 (PBST). For the *A. fumigatus* biofilm adherence assay, Sabouraud media was used, while for induction of *alcA* promoter, fungal strains were grown in *Aspergillus* minimal medium (AMM) [22] (0.152% KH_2_PO_4_, 0.052% KCl, 0.6% NaNO_3_, 0.05% MgSO_4_, 1 ml/l trace elements, 1% (v/v) EtOH, 2% Lactose, 0.05% Glucose, pH 6.5). For observation of conidia pigmentation, conidia were grown on Sabouraud agar for 6 days at 37°C. To study fungal hyphal growth, 10^6^ conidia were used to inoculate the center of YPD plates and incubated at 37°C. Colony diameter was measured daily.

### Amino Acid and Nucleic Acid Sequences

The 683 amino acid sequence of MedA (GenBank: EAL93620.1) and the amino acid sequences of MedA orthologues, i.e. MedA from *A. nidulans* (GenBank: AAC31205.1), ACON-3 from *N. crassa* (GenBank: ADL28820.1), Acr1 from *M. grisea* (GenBank: BAC41196.1), and Ren1 from *F. oxysporum* (GenBank: BAC55015.1), were obtained from the National Center for Biology Information (www.ncbi.nlm.nih.gov). Identification of the conserved region in MedA was performed using BLASTp search of the NCBI Reference Proteins (refseq_protein) database, and the *Aspergillus fumigatus* MedA 683 amino acid sequence as a query sequence after restricting the BLAST search to fungi only. To ensure best accuracy of the BLAST output, amino acid sequences of hypothetical proteins annotated as MedA-homologous proteins were selected, neglecting hypothetical and incomplete proteins, as well as those not annotated as MedA-homologous proteins. MedA and MedA- homologous proteins were then aligned using ClustalX multiple alignment application [23]. Prediction of the NLS1 motif for MedA was performed using PSORT II (psort.hgc.jp/form2.html) [24]. DNA-binding motif searches were performed using DNA-binding protein prediction server DNABIND (www.enzim.hu/~szia/dnabind.html) [25] and BLASTp search of the NCBI. MedA secondary structure analysis was performed using HHpred [26] and Jpred3 [27], and fold prediction using PHYRE [28]. Query results in DSSP format were manually aligned against the MedA amino acid sequence and compared.

**Figure 1:**
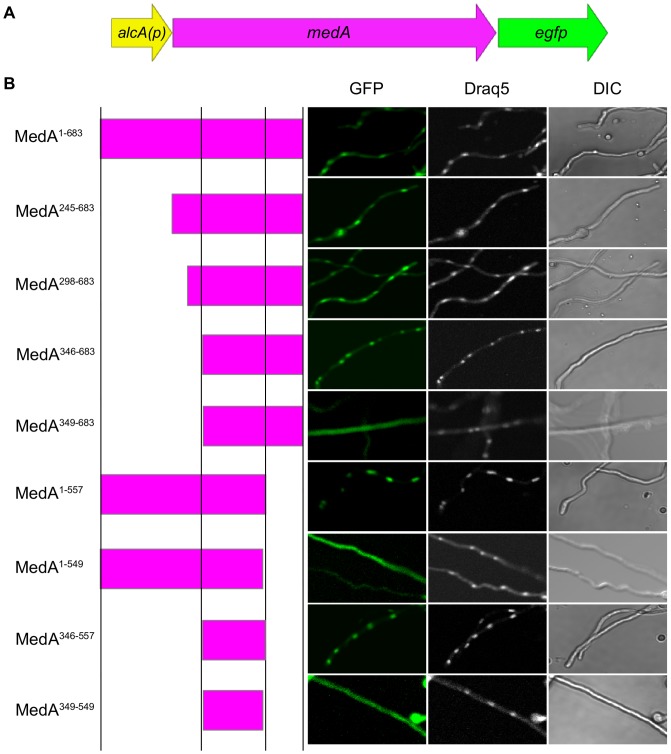
Subcellular localization of the MedA-EGFP fusion truncation constructs in *A. fumigatus*. (A) Schematic overview of the various truncated *medA-egfp* gene fusions. Full-length *medA* and different *medA* truncations were fused in-frame to *egfp* under the control of *alcA* promoter. (B) The cellular localization of various MedA-EGFP fusion proteins expressed in *A. fumigatus* Af293. Nuclei were stained by Draq5 and mycelia were analyzed by light microscopy. Left, center, and right columns show the GFP, Draq5, and DIC (Differential Interference Contrast), respectively. The MedA-GFP constructs are indicated on the left side. Vertical lines, from left to right, represent amino acids 1, 346, 557, and 683 respectively.

### RNA Extraction and RT-PCR

Conidia were grown at 37°C for 24 hr in Sabouraud medium and RNA was extracted using NucleoSpin® RNA Plant kit (Macherey-Nagel GmbH & Co. KG) according to the manufacturer instructions. cDNA synthesis was performed using the QuantiTect Reverse Transcription Kit (Qiagen). MedA gene expression was measured by quantitative real-time RT-PCR using the fluorescent reporter SYBR Green (Fermentas) and ABI 7300 thermocycler (Applied Biosystems). RT-PCR was performed for *medA* using primer pair RT-MedA-F and RT-MedA-R. The endogenous reference gene, *tef1*, was quantified using the primer pair TEF1-sense and TEF1-antisense [17]. Quantification of mRNA level of *medA* was performed using 2^–ΔΔCt^ method [29], [30].

### Construction of GFP-tagged MedA Truncations

The *medA* open reading frame was PCR amplified from genomic DNA using the primers MedA-F and MedA-R and then cloned into plasmid pGFP-Phleo [31] using EcoRV and NotI, resulting in plasmid pMedA-GFP. Next, the *alcA* promoter was amplified from plasmid pAL4 by PCR using the primers AlcAp-For and AlcAp-Rev and used to replace the *gpdA* promoter in the plasmid pMedA-GFP after AgeI and EcoRV digestion. The resulting plasmid was designated pAlcA-MedA-GFP. Individual *medA* truncations were amplified by PCR and cloned into the plasmid pAlcA-MedA-GFP using EcoRV and NotI, replacing the full-length *medA* gene. The constructed plasmids, the combinations of oligonucleotides used to clone these plasmids, and the corresponding generated *A. fumigatus* strains are listed in Table 2.

**Figure 2:**
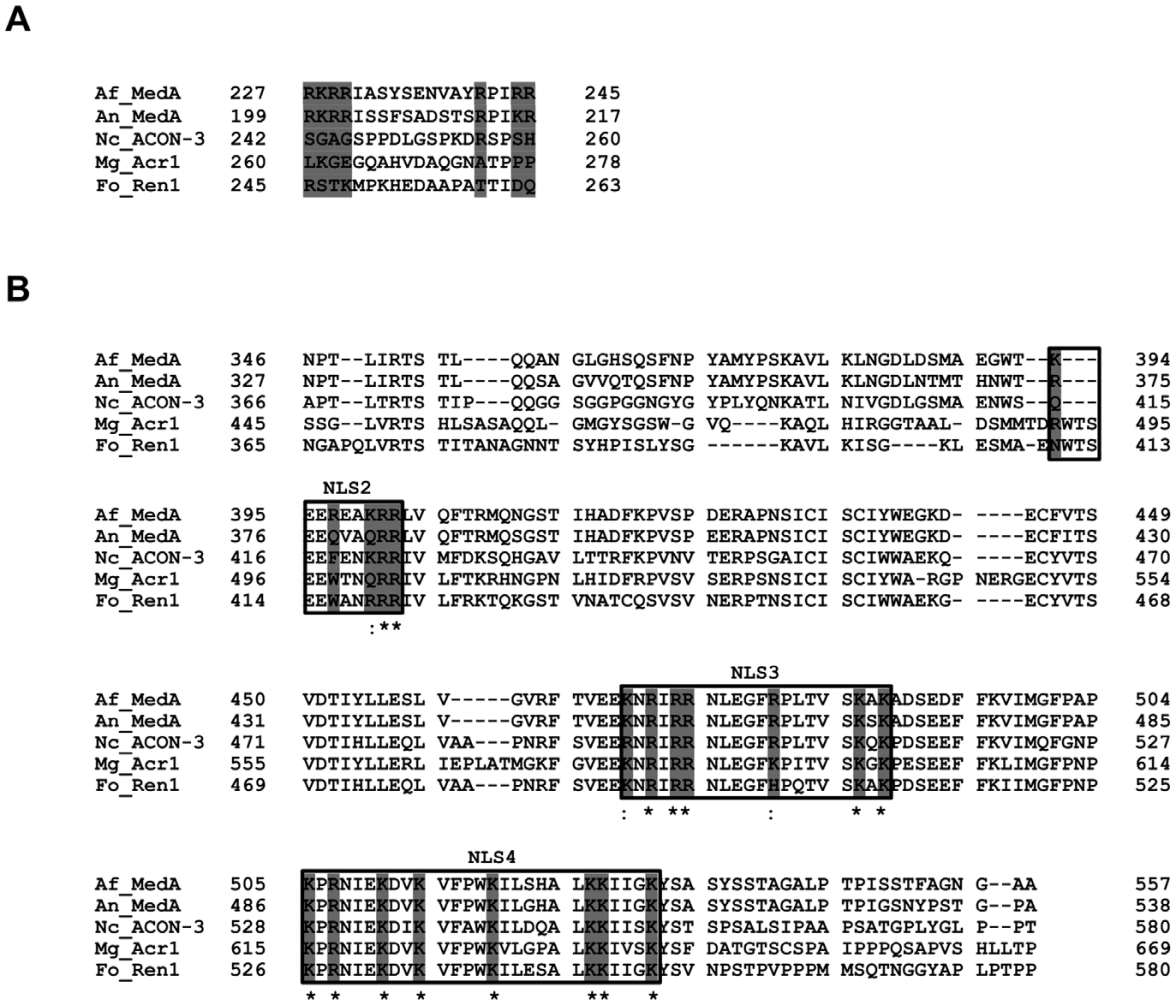
Amino acid sequence alignment of *A. fumigatus* MedA putative NLSs 1–4 with other MedA homologues. (A) Sequence alignment and motif prediction using PSORT II identified NLS1 sequence among orthologues of MedA. (B) Sequence alignment of the MedA minimal nuclear localization domain, MedA^346–557^ with other MedA orthologues. The sequences representing the putative NLSs 2, 3, and 4 are boxed. The basic amino acids within the putative NLSs of *A. fumigatus* MedA and the corresponding amino acids in MedA orthologues are highlighted in gray. The presence of an asterisk or a colon below the basic amino acids indicates a fully or strongly conserved residue, respectively. Numbers indicate the amino acid position within the primary amino acid sequence of the protein. Af_MedA: *A. fumigatus* MedA (GenBank: EAL93620.1), An_MedA: *A. nidulans* MedA (GenBank: AAC31205.1), Nc_ACON-3: *N. crassa* ACON-3 (GenBank: ADL28820.1), Mg_Acr1: *M. grisea* Acr1 (GenBank: BAC41196.1), and Fo_Ren1: *F. oxysporum* Ren1 (GenBank: BAC55015.1).

### Site-directed Deletion Mutagenesis of MedA Putative Nuclear Localization Signals

Deletion of NLS1, NLS2, and NLS3 of *medA* was done by fusion PCR [32]. Briefly, *medA* was amplified from the plasmid pAlcA-MedA-GFP using the primers MedA-F and MedA-R while deletions spanning NLS1, NLS2, and NLS3 in *medA*, i.e. *medA^ΔNLS1^*, *medA^ΔNLS2^*, and *medA^ΔNLS3^*, were obtained using the primer pairs that carry the desired deletion mutation (Table 2). The PCR products were then ligated into the EcoRV-NotI site of plasmid pAlcA-MedA-GFP to replace the intact *medA* gene as above. Deletion of NLS4 in *medA*, designated *medA^ΔNLS4^*, was achieved by PCR amplification of 1518 bp *medA* fragment using the primers MedA-F and NLS4-R. The PCR product was then cloned into pAlcA-MedA-GFP using NheI and SnaBI, generating plasmid pMedA-GFP-ΔNLS4. The constructed plasmids and the corresponding *A. fumigatus* expression strains are listed in Table 2.

### Construction of Expression Plasmids for *medA*, *medA^ΔNLS1^*, *medA^ΔNLS2^*, *medA^ΔNLS3^*, and *medA^ΔNLS4^* under the Expression of the Endogenous Promoter of *medA*

Complementation of Δ*medA* phenotype studies were performed using strains expressing *medA*, *medA^ΔNLS1^, medA^ΔNLS2^*, *medA^ΔNLS3^*, and *medA^ΔNLS4^* under the control of the endogenous 1.5 kb *medA* promoter, *medA(p)*. To generate these strains, the 1.5 kb promoter region and the first 282 bp of *medA* coding sequences was amplified from *A. fumigatus* genomic DNA using the primers PmedA-F and MedA-NheI-R. This PCR product was digested with NheI and NruI and subcloned upstream of the *medA* open reading frame of the pMedA-GFP plasmid, after NheI and EcoRV digestion. The resulting plasmid was designated pPmedA-MedA-GFP. The *medA(p)* sequences were released from this plasmid by NheI and AgeI digestion and used to replace the *alcA* promoter upstream sequences of *medA* in pMedA-GFP-ΔNLS1, pMedA-GFP-ΔNLS2, pMedA-GFP-ΔNLS3, and pMedA-GFP-ΔNLS4; producing plasmids pPmedA-MedA-GFP-ΔNLS1, pPmedA-MedA-GFP-ΔNLS2, pPmedA-MedA-GFP-ΔNLS3, and pPmedA-MedA-GFP-ΔNLS4, respectively. The *A. fumigatus* Δ*medA* mutant was then transformed with these plasmids. The plasmids and the corresponding *A. fumigatus* expression strains are listed in Table 2.

**Figure 3:**
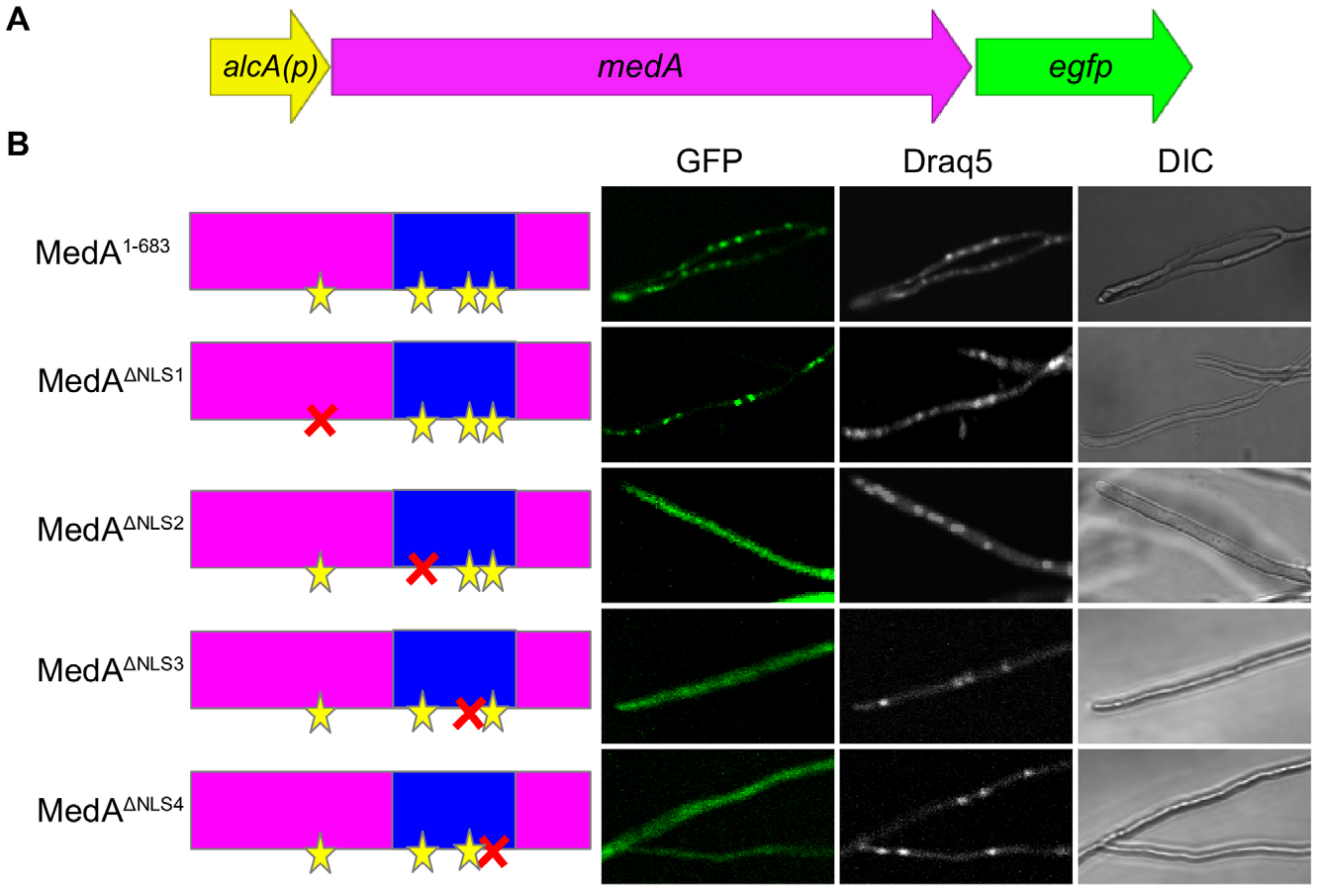
Deletion of putative NLS sequences in *A. fumigatus* MedA. (A) Schematic overview of the *medA-egfp* gene fusions expressed by *alcA* promoter in the Af293 wild type strain. (B) The effect of deletion of the MedA NLS sequences on the subcellular localization of MedA-EGFP fusion proteins in *A. fumigatus* Af293. Nuclei were stained by Draq5 while mycelia were analyzed by light microscopy. Left, center, and right columns show the GFP, Draq5, and DIC (Differential Interference Contrast), respectively. The names of the deleted NLS and the depiction of the different MedA-EGFP fusion proteins are indicated on the left side. The putative NLSs 1–4 are indicated by yellow stars while the deleted NLS is indicated by red X. The minimal nuclear localization domain, MedA^346–557^ is indicated by the blue box.

### Generation of *A. fumigatus* Strain that Encodes *medA^346–557^* under the Expression of the *medA* Endogenous Promoter

The plasmids, pPmedA-MedA(1036–1671), which encodes *medA^346–557^*, was constructed using fusion PCR [32]. The 1.5 kb promoter region of *medA* and the 636 bp *medA^346–557^* region were PCR amplified using the primers PmedA-AgeI-F and MedA-1671-R while the primer pairs PM(1036)-R and PM(1036)-F provided the 5' complementary sequences for hybridization. The two PCR products were hybridized and digested with NheI and NruI to replace the *gpdA* promoter of the pGFP-phleo plasmid, which was digested with NheI and the compatible blunt end EcoRV. The plasmid was used to transform the Δ*medA* strain and the resulting strain was designated ΔM-M(346–557).

### Fungal Biofilm Adherence Assay

Fungal biofilm adherence assay was performed using 6-well non-tissue culture-treated plates as described previously [33]. Wells were inoculated with 1 ml of Sabouraud media containing 10^5^ conidia and incubated for 24 hr at 37°C. Biofilms were washed 3 times with 3 ml of PBS containing Ca & Mg (Thermo Scientific) and stained with 3 ml 0.05% (w/v) crystal violet solution for 24 hr.

### Hydrophobicity Assay of Conidia

Aerial conidia were collected with a cotton swab from cultures grown for 6 days on YPD agar. Conidia were then resuspended in 3 ml mineral oil-water mix. The conidia located in oil phase were considered hydrophobic while conidia resuspended in water were considered hydrophilic.

**Figure 4:**
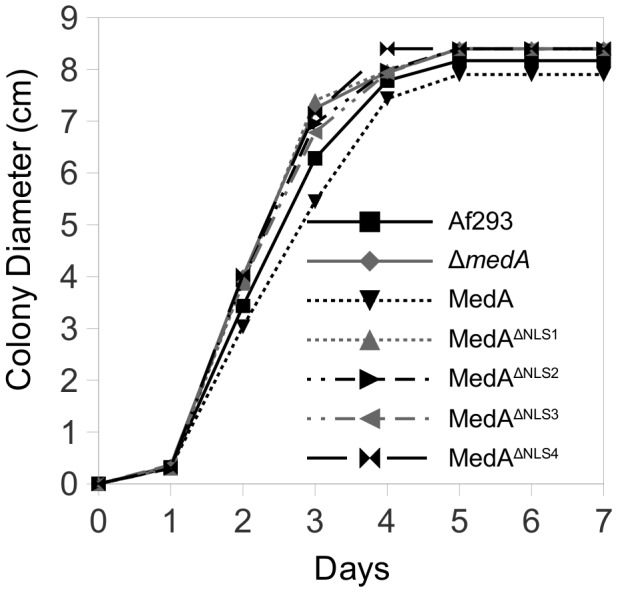
Mycelial growth of *A. fumigatus* is not affected by MedA nuclear localization. YPD agar plates were spot inoculated with the indicated strains and the colony diameter measured daily. Af293 is the *A. fumigatus* wild type strain; MedA, MedA^ΔNLS1^, MedA^ΔNLS2^, MedA^ΔNLS3^, and MedA^ΔNLS4^ indicate expression of the corresponding construct under the control of the *medA(p)* in the Δ*medA* strain.

### Fluorescence Microscopy

*A. fumigatus* conidia were grown in liquid AMM for 48 hr at 37°C. Slides were prepared by mixing 20 µl of sample with 0.5 µl of 5 mM Draq5. Subcellular localization studies were performed under a confocal laser scanning microscope (x40) Fluoview FV1000 (Olympus, Tokyo, Japan) equipped with 488 nm and 633 nm lasers for GFP and Draq5 excitation, respectively. Images were processed for optimal presentation using GIMP (GNU Image Manipulation Program) software.

### Survival Assay

Virulence was tested using *Galleria mellonella* as described previously [17], [34]. Sixth instar of *G. mellonella* larvae, were injected with 10 µl of 10^7^ swollen conidia ml^−1^, by insertion of a Hamilton needle through the last pseudopod. Uninfected worms were sham infected with 10 µl of YPD media. After injection, worms were incubated at 37°C in the dark and surviving worms were counted daily. The Wilcoxon sum test was used to test for significant differences in survival between groups.

**Figure 5:**
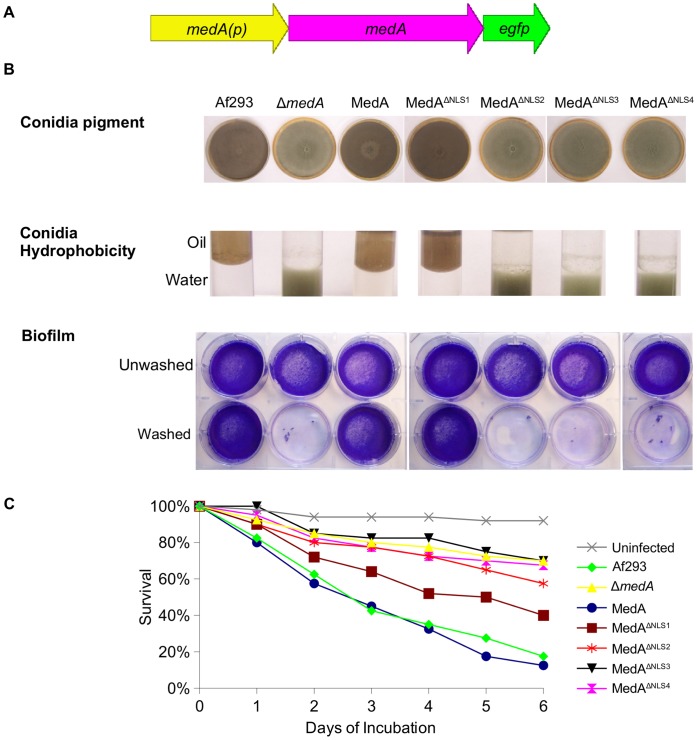
Effect of MedA nuclear localization on restoring wild type phenotype. (A) Schematic overview of the *medA-egfp* fusion constructs under the control of the 1.5 kb *medA* promoter, *medA(p)* used for complementation of the Δ*medA* strain. (B) Vegetative growth of strains on Sabouraud agar for 6 days at 37°C; hydrophobicity of conidia; and biofilm formation before and after washing. (C) Survival assay of *G. mellonella* larvae. 40 worms/strain were infected with 10^5^ swollen conidia. Af293 indicates the *A. fumigatus* wild type strain; MedA, MedA^ΔNLS1^, MedA^ΔNLS2^, MedA^ΔNLS3^, and MedA^ΔNLS4^ indicate expression of the corresponding construct under the control of the *medA(p)* in the Δ*medA* strain. Virulence of strains expressing cytoplasmic and nuclear MedA was compared to Af293 and Δ*medA* strain, respectively, using the log rank test. For all comparisons, P was ≤0.05.

## Results

### MedA Nuclear Localization is Mediated via Sequences within the Conserved C-terminal Domain

Previous analyses have demonstrated that MedA in hyphae and conidiophores of *A. nidulans* is located predominately within the nucleus, however the sequences governing nuclear localization remain unknown [20]. To identify candidate regions within MedA that might govern nuclear localization, a BLAST and alignment analysis of MedA orthologues was performed. This analysis revealed the presence of a highly conserved region within the C-terminal half of MedA (aa 346–565). The precise length of the conserved region varied in length depending on the species used for comparison (data not shown). In light of this conservation of sequence, we hypothesized that this region might contain sequences essential for nuclear localization and subsequent function of MedA.

To investigate the role of this domain in mediating MedA nuclear localization, we generated a series of GFP-tagged MedA truncations constructs, and expressed these constructs in the *A. fumigatus* wild type strain Af293 under the control of the inducible *A. nidulans alcA* promoter (Figure 1A). Consistent with reports of nuclear localization in other MedA orthologues, induction of the intact *A. fumigatus medA-gfp* expression construct resulted in a predominant accumulation of GFP within the nucleus (Figure 1B). Deletion of up to 345 N-terminal amino acids of MedA did not affect the nuclear localization of MedA, suggesting that these sequences are dispensable for nuclear localization (Figure 1B). Extending this deletion to include the first 348 amino acids resulted in predominately cytoplasmic accumulation of the MedA-GFP construct (Figure 1B). Similarly, while deletion of the C-terminal amino acids 558–683 had no effect on nuclear localization, extending this deletion to begin at amino acid 550 resulted in a strain with predominately cytoplasmic accumulation of MedA-GFP (Figure 1B). Collectively, these results suggest that the nuclear localization domain(s) of MedA lie between amino acid 346 and 557. To confirm these results, we constructed an expression constructs in which GFP was fused to this putative minimal nuclear localization domain identified above (aa 346–557) and examined its subcellular distribution. As predicted, this domain alone was sufficient to mediate nuclear localization while decreasing the size of this construct minimally (encompassing amino acids 349–549 of MedA) resulted in a construct that remained predominately cytoplasmic (Figure 1B). Of note, the MedA^346–557^ fragment which localizes to the nucleus spans the conserved sequences identified by sequence alignment of the MedA orthologues. Interestingly, although the most highly conserved region identified in this alignment was also contained within the smaller MedA^349–549^ fragment, this construct failed to localize to the nucleus (Figure 1B). Thus, other sequences immediately bordering the core conserved region are likely required for normal nuclear localization or maintaining normal protein structure.

**Figure 6:**
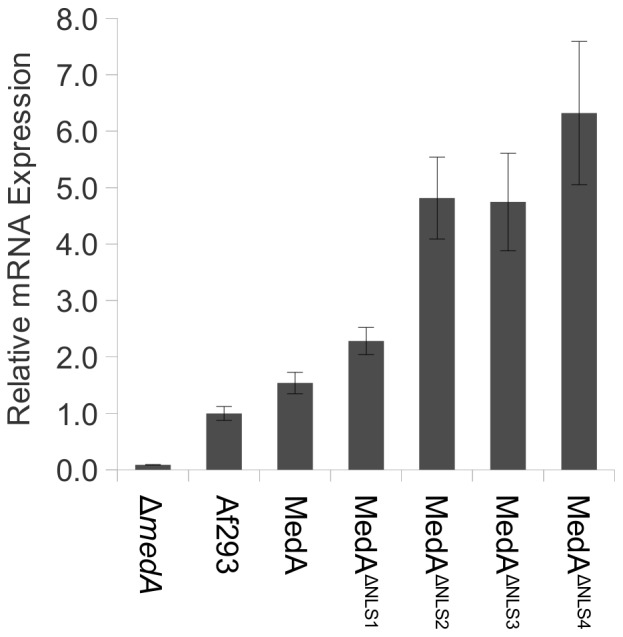
Relative gene expression of cytoplasmic and nuclear *medA* measured by RT-PCR. Af293 is the *A. fumigatus* wild type strain; MedA, MedA^ΔNLS1^, MedA^ΔNLS2^, MedA^ΔNLS3^, and MedA^ΔNLS4^ indicate expression of the corresponding construct under the control of the *medA(p)* in the Δ*medA* strain, normalized to *medA* expression in strain Af293. Error bars represent the standard error of three triplicates for every strain.

Additionally, differences in the intensity of nuclear staining were evident between the various constructs. Full-length MedA, and the C-terminal deletion constructs MedA^1–557^, and MedA^1–549^ required two days of *alcA* induction for high level fluorescence to be evident. In contrast, all MedA N-terminal truncations studied showed a strong fluorescence after only one day of induction, suggesting that N-terminal sequences of MedA might contain sequences important for the regulation of MedA nuclear expression levels.

### MedA Requires the Entire Nuclear Localization Domain for Nuclear Localization

Importin-mediated nuclear localization is classically facilitated by cluster(s) of positively charged amino acids, i.e. lysine (K) and arginine (R). These clusters may be present singly (monopartite) or duplicate (bipartite) in which case they are classically separated by 10–12 amino acids [35], [36].

To identify possible nuclear localization signal (NLS) sites in MedA, we performed a sequence analysis of the entire MedA amino acid sequence using PSORT II. The analysis predicted the presence of a putative nuclear localization signal, designated NLS1. However, NLS1 was located outside the 212 aa minimal nuclear localization domain, MedA^346–557^, and was not conserved when was aligned with other MedA orthologues (Figure 2A). To identify other possible non-canonical NLS regions, we performed a sequence alignment of the minimal nuclear localization domain of MedA^346–557^, with ACON-3 from *N. crassa*, Acr1 from *M. grisea*, Ren1 from *F. oxysporum*, and MedA from *A. nidulans*. This region was observed to be rich in positively charged residues, containing 29 K and L residues among its 212 amino acids (∼13.5%). Among these, 19 were fully conserved in all five studied MedA orthologues, and 4 were partially conserved in that all exchanges were either K to R or R to K, (Figure 2B). Although this region lacked classical NLS sequence, 3 sets of sequences rich in positively charged residues were identified and designated NLS2, NLS3, and NLS4. NLS2 contains sequences consistent with the monopartite class, while NLS3 and NLS4 are complex regions containing elements consistent with both monopartite and bipartite classes. NLS3 contains a monopartite cluster of KNRIRR and a bipartite two clusters of RR and KAK separated by 11 amino acids. NLS4 is composed of a bipartite cluster surrounded by a number of R and K residues within 6 amino acids of each cluster (Figure 2B).

**Figure 7:**
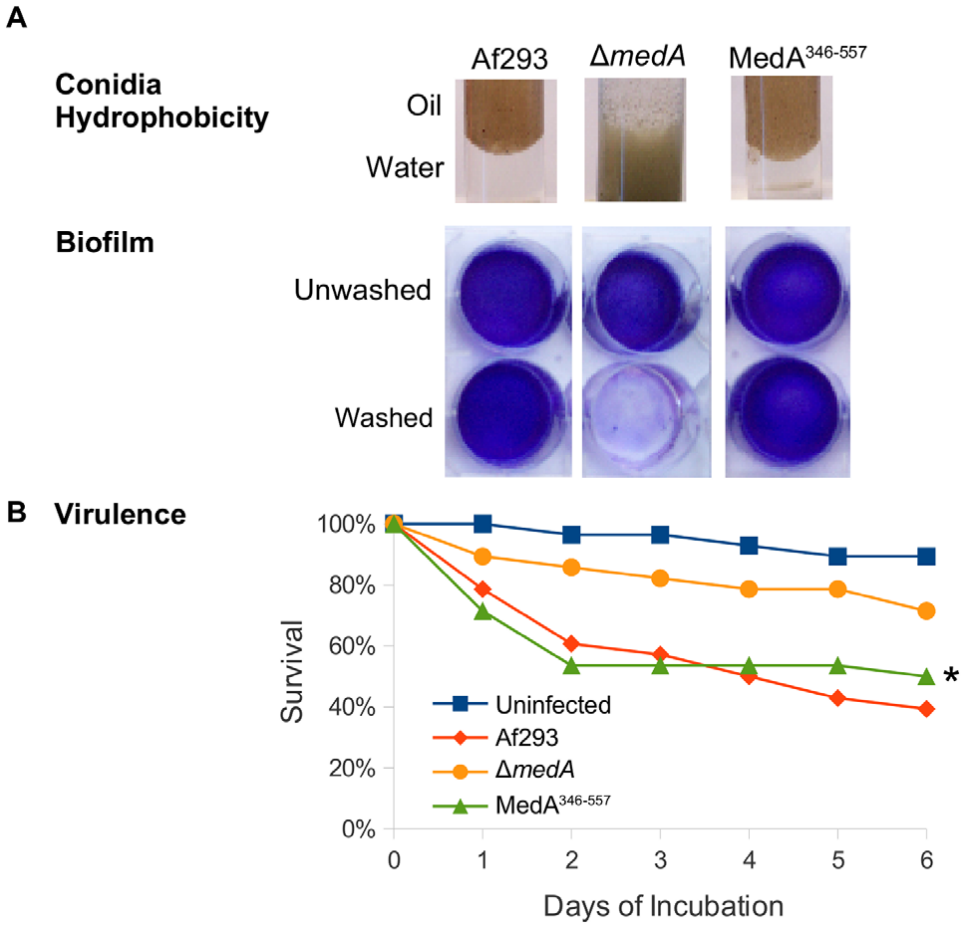
Phenotypic analysis of Δ*medA* strain expressing MedA^346–557^ domain. (A) Conidia hydrophobicity and biofilm formation of the indicated strains. (B) Survival assay of *G. mellonella* larvae. 28 worms/strain were infected with 10^5^ swollen conidia. Af293 indicates the *A. fumigatus* wild type strain; MedA^346–557^ indicates expression of this construct under the control of the *medA(p)* in the Δ*medA* strain. Analysis of survival data was performed using the log rank test. Statistically significant differences are indicated by asterisk (P value ≤0.05).

To investigate the role of NLS1, NLS2, NLS3, and NLS4 in nuclear localization, a functional analysis of the four NLSs was performed. Site-directed deletion mutagenesis of NLS1, NLS2, NLS3, and NLS4 of MedA was performed, generating constructs *medA^ΔNLS1^*, *medA^ΔNLS2^*, *medA^ΔNLS3^*, and *medA^ΔNLS4^* which were expressed in *A. fumigatus* Af293 under the inducible *alcA* promoter. The effect of mutagenesis on MedA-GFP subcellular localization was then determined using confocal microscopy. As predicted from the truncation studies, the deletion of NLS1 did not affect the nuclear localization of MedA (Figure 3B). Surprisingly, however, deletion any one of the three other NLS abrogated the nuclear localization of MedA (Figure 3B). Importantly, expression of the cytoplasmic MedA^ΔNLS2^, MedA^ΔNLS3^, or MedA^ΔNLS4^ with wild type MedA in the *A. fumigatus* Af293 strain did not affect adherence, conidiation or conidial pigment (data not shown) suggesting that these constructs do not result in dominant negative effects. Collectively, these results suggest that MedA nuclear localization is facilitated by the entire nuclear localization domain, rather than any single NLS.

### MedA Nuclear Localization is Required for Adherence, Conidiation and Virulence in *A. fumigatus*

Previous studies revealed that MedA plays an essential role in conidial hydrophobicity, mycelial adherence to substrates, and fungal pathogenicity [17]–[19]. We therefore tested the role of nuclear localization of MedA in mediating these phenotypes using the NLS deletion mutant constructs of MedA. For these studies, a 1.5 kb region upstream of the *medA* ORF encompassing the predicted promoter sequences was cloned upstream of each of the *medA^ΔNLS^* constructs. These constructs were then expressed in the Δ*medA* mutant strain, and the resulting phenotypes were assayed.

As predicted, no difference in hyphal growth between wild type *A. fumigatus*, the Δ*medA* mutant, or any of the MedA construct complemented strains was observed, regardless of their subcellular localization (Figure 4). In contrast, nuclear localization of MedA was required for normal conidia development. Conidia obtained from the Δ*medA* strain and Δ*medA* strains expressing MedA constructs with cytoplasmic localization (cytoplamic MedA) were bright green, and markedly hydrophilic, while all strains expressing MedA which localized to the nucleus (nuclear MedA) phenocopied the *A. fumigatus* wild type strain and produced hydrophobic grey-green conidia. Similarly, MedA nuclear localization was required for the formation of adherent fungal biofilms, while strains expressing cytoplasmic MedA formed non-adherent mycelia mats similar to those formed by the Δ*medA* mutant (Figure 5B). Finally, the virulence of the mutant strains in an invertebrate model was also found to be dependent on the subcellular localization of MedA. *Galleria* larvae infected with either Δ*medA* strain or Δ*medA* strains complemented with cytoplasmic MedA survived longer compared with those infected with either *A. fumigatus* wild type strain, or strains expressing nuclear MedA (Figure 5C).

Importantly, the failure of the cytoplasmic MedA constructs to complement the *medA* mutant was not likely a consequence of inadequate expression, as real time RT-PCR revealed that cytoplasmic MedA strains produced higher levels of *medA* mRNA than did nuclear MedA expressing strains (Figure 6). Collectively these results suggest that nuclear localization is required for the function of MedA.

### Expression of *medA^346–557^*, Encoded by the *medA* Endogenous Promoter, is Sufficient to Complement the Function of MedA

The results of our truncation analysis suggested that amino acids 346–557 are sufficient to mediate nuclear localization of MedA. To determine if this region is also sufficient to generate functional MedA, or if additional upstream or downstream sequences are required to mediate MedA dependent phenotypes, we tested the ability of MedA^346–557^ to complement the Δ*medA* mutant. This construct was expressed under the control of *medA* endogenous promoter as described above and expressed in the Δ*medA* mutant strain. Complementation of the Δ*medA* mutant strain with the MedA^346–557^ domain restored the wild type phenotype with respect to conidiation, conidial hydrophobicity, the formation of adherent biofilms and the virulence in invertebrate model (Figure 7A–B). Thus, the minimal nuclear localization fragment (MedA^346–557^) is both necessary and sufficient to govern conidiation, adherence and virulence in *A. fumigatus*.

## Discussion

MedA is a developmental regulator that governs the expression of diverse biological processes in filamentous fungi [37], [38]. In order to begin to unravel the mechanism by which this protein regulates development and virulence, we performed a deletion analysis of MedA in *A. fumigatus*. These studies demonstrate that nuclear localization of MedA in *A. fumigatus* is mediated by a C-terminal domain (MedA^346–557^) conserved among MedA orthologues. Surprisingly, this region contained no canonical NLS sequences. Indeed, a bioinformatic analysis of *A. fumigatus* MedA using PSORT II revealed the presence of a single NLS sequence (NLS1) that was located outside of the conserved domain MedA^346–557^. However, NLS analysis tools utilize the protein primary amino acid structure for prediction, and are therefore limited in their ability to predict NLS sequences arising from protein folding and three dimensional architecture [39]. We therefore used direct sequence inspection and site-directed mutagenesis to identify and tested candidate sequences mediating nuclear localization [40], [41]. This approach identified three regions rich in basic amino acids, although none of these sequences completely matched classical mono- or bipartite NLS patterns. Interestingly, deletion of any one of these three regions was sufficient to impair nuclear localization, suggesting either a cooperative function in mediating nuclear import, or an important role for these sequences in preserving the three dimensional structure of this MedA domain. Given that these regions contained over 29 conserved K/R amino acids, single amino acid mutagenesis to define the exact role of each of these residues in nuclear import is impractical.

Our findings demonstrated a clear relationship between nuclear import and the function of MedA since nuclear localization of MedA was required for the complementation of Δ*medA* phenotype. The failure of the cytoplamic MedA constructs to complement the *medA* mutant was not a consequence of inadequate expression since quantitative real time RT-PCR analysis revealed that cytoplasmic MedA strains produced higher levels of *medA* mRNA than did the nuclear MedA expressing strains, and the degree of fluorescence exhibited by these strains was higher than that observed with strains expressing nuclear MedA. In addition, over-expression of the cytoplasmic MedA^ΔNLS2^, MedA^ΔNLS3^, or MedA^ΔNLS4^ in the *A. fumigatus* wild type strain Af293 under the inducible *alcA* promoter did not result in a MedA deficient phenotype, arguing that a dominant negative effect of cytoplasmic MedA constructs is unlikely [42]. These results are most consistent with MedA playing a role in governing gene expression either directly, or indirectly. The conserved MedA^346–557^ domain was not only sufficient to mediate nuclear localization, but also to recover the *A. fumigatus* wild type phenotype when expressed in the Δ*medA* background. Thus, it is likely that this region contains not only the nuclear localization signal, but also the necessary sequences to govern gene expression either through directly binding DNA or interacting with other proteins. A bioinformatics analysis of MedA^346–557^ using DNABIND [25] and NCBI BLAST revealed no DNA-binding domain in MedA, although it is possible that MedA^346–557^ contains novel amino acid sequences for the recognition of DNA. A more plausible hypothesis however, is that MedA^346–557^ functions through interaction with other regulatory proteins. MedA^346–557^ contains 13 glutamatic acid and 9 aspartic acid within its 212 amino acid sequence. These two negatively charged polar amino acids are frequently involved in protein active or binding sites [43]. This protein interaction hypothesis is also supported by the fact that the secondary structure of this region, analyzed using HHpred and Jpred3, is predicted to be composed of numerous alpha helices and beta strands which are necessary for protein architecture. Unfortunately, homology modeling tools such as PHYRE failed to identify robust structural similarities between MedA and other known proteins, limiting this bioinformatic approach. Future studies focused on identifying the MedA-protein interactome may help better characterize the exact function of MedA^346–557^.

Despite the clear ability of MedA^346–557^ to complement the Δ*medA* phenotype, a role for the N- and C-terminal amino acids upstream and downstream of MedA^346–557^ in MedA function should not be discounted. Our study was limited in that only MedA dependent conidial hydrophobicity, biofilm adherence and virulence in invertebrates were tested. It remains possible that the N- and C- terminal domains of MedA are required for the regulation of other MedA-dependent biological processes that were not examined. Further, these sequences may play an important regulatory role in the expression or kinetics of MedA, that was not apparent in our static assays. Indeed, the observations that full-length MedA and the C-terminal deletion constructs MedA^1–557^, and MedA^1–549^ required two days of *alcA* induction for high level fluorescence while N-terminal deletions produced visible fluorescence after only 24 hours might suggest that the N-terminal sequences of MedA play a role in protein stability or degradation.
